# Clinical characteristics and prognostic implications of BRCA-associated tumors in males: a pan-tumor survey

**DOI:** 10.1186/s12885-020-07481-1

**Published:** 2020-10-14

**Authors:** Peng Sun, Yue Li, Xue Chao, Jibin Li, Rongzhen Luo, Mei Li, Jiehua He

**Affiliations:** 1 State Key Laboratory of Oncology in South China, Collaborative Innovation Center for Cancer Medicine, Guangzhou, P. R. China 510060; 2 Department of Pathology, Sun Yat-sen University Cancer Center, Guangzhou, P. R. China 510060; 3 Department of Pathology, Sun Yat-sen University Cancer Center, 651 Dongfeng East Road, 510080 Guangzhou, P. R. China; 4 Department of Molecular Diagnosis, Sun Yat-sen University Cancer Center, Guangzhou, P. R. China 510060; 5 Department of Clinical Research, Sun Yat-sen University Cancer Center, Guangzhou, P. R. China 510060

## Abstract

**Background:**

The BRCA mutation (BRCAm) in males has been reported to confer a higher risk for the development of various tumors. However, little is known about its clinicopathologic features and prognostic implications.

**Design:**

We conducted a retrospective pan-tumor survey on 346 cases of BRCA-associated tumors in males. Comparative analyses were conducted among male and female patients with BRCAm (*n* = 349), as well as in male patients without BRCAm (*n* = 4577).

**Results:**

Similar incidences of BRCAm (6.0 vs. 6.6%) and age at diagnosis of tumor (median, 65 vs. 60 years) were observed in male and female patients. Carcinomas of the lung, bladder, stomach, and cutaneous melanoma were the frequent tumors demonstrating BRCAm in males, of which the majority were stage II or III diseases with a higher frequency of BRCA2 mutations. Compared to that in the non-BRCAm group, cutaneous melanoma (16.3 vs. 5.0%), lung cancer (19.4 vs. 11.8%), bladder cancer (15.6 vs. 5.6%), and stomach cancer (11.9 vs. 5.5%) accounted for a higher proportion in the BRCAm group. Advanced disease and more mutation counts (median, 322 vs. 63 mutations) were also found in the BRCAm group. A total of 127 BRCA1 and 311 BRCA2 mutations were identified, of which 21.8 and 28.6% were deleterious, respectively. Frequent deleterious variants were identified in carcinomas of the breast (100.0%), colorectum (62.2%), prostate (43.3%), and stomach (42.9%). BRCA1 fusions with NF1, FAM134C, BECN1, or LSM12 and recurrent BRCA2 mutations at P606L/S, E832K/G, and T3033Lfs*29 were detected. Frameshift mutations in BRCA2 at N1784 (N1784Kfs*3, N1784Tfs*3) were frequently observed in both male and female patients. Compared with those in females, BRCA mutations in males were associated with decreased overall survival (OS) and progression-free survival (PFS). Male patients with deleterious BRCAm displayed increased OS compared with non-BRCAm carriers. The subgroup analysis demonstrated that BRCAm was associated with increased OS in gastric and bladder cancers, decreased PFS in prostate, esophageal, and head and neck cancers, and decreased OS in glioma/glioblastoma in males.

**Conclusion:**

These findings provide an overview of the distinct characteristics and clinical outcomes of male patients with BRCA-associated tumors, suggesting the importance of further genetic BRCA testing in males.

## Background

Breast cancer susceptibility gene (BRCA), including BRCA1 and BRCA2, are involved in the homologous recombination repair of DNA double-strand breaks [1, 2]. As tumor suppressor genes, loss-of-function mutations in BRCA1/2 may lead to the accumulation of DNA double-strand breaks and result in genomic instability and tumor formation [1, 2]. Despite the general nature of BRCA functions, female BRCA1/2 mutation (BRCAm) carriers predominantly display an increased risk of breast and ovarian cancers [3–5]. The incidences and clinical characteristics of melanoma, pancreatic cancer, colorectal cancer, and other tumors in female BRCA1/2 carriers have been examined with conflicting results [6–8].

In addition, increasing concerns have been raised regarding the importance of BRCA1/2 testing in males. Men carrying mutations in BRCA1/2 have been reported to develop melanomas [6] or pancreatic [7], prostatic [9], and breast cancers [10]. Moreover, much clinical evidence [11–15] indicates that the germline or somatic inactivation of BRCA1/2 in male patients with malignancy confers sensitivity to DNA damaging therapies, such as poly ADP-ribose polymerase inhibitor (PARPi) [16, 17]. However, in both healthy individuals and patients with malignancy, far fewer men than women are currently tested for mutations in these genes. The clinical characteristics and prognostic implications of BRCA-associated tumors in males are also underdetermined.

To address these issues, we retrospectively reviewed male BRCAm carriers with a diagnosis of tumor from our cancer genetics database (SYSUCC cohort, *n* = 52) and a public database (TCGA cohort, *n* = 294). Pan-tumorous BRCA1/2 mutational profiles were described and compared between male and female patients (TCGA cohort, *n* = 349). Comparative analyses of clinicopathologic features and prognostic implications were conducted among male and female patients with BRCAm, as well as male patients without BRCAm (TCGA cohort, *n* = 4577). The present study aimed to provide a resource for investigating the contribution of BRCA related to the clinical characteristics and prognostic implications of tumors in males.

## Methods

### Data collection for the SYSUCC cohort

Male patients with germline or somatic BRCA1/2-associated tumors (*n* = 52) diagnosed between January 2012 and December 2019 were identified from the genetics database of Sun Yat-sen University Cancer Center (SYSUCC). Patients with deleterious or likely deleterious BRCA1/2 mutation as well as those with variants of undetermined significance were included in the cohort.

The pathogenicity of the BRCA mutation was also individually verified for concordance using public databases, including OncoKB and ClinVar. Clinicopathologic data, including age at diagnosis, family history of tumor, diagnosis of multiple tumors, anatomic site of the tumor, histology, histologic grade, TNM stage, microsatellite instability (MSI), tumor mutation burden (TMB), surgical procedures, systemic chemotherapy, radiotherapy, and survival data, were recorded. All relevant data were extracted by two independent reviewers (PS and YL), and any disagreements were reviewed by a third reviewer (JHH). This study was conducted in accordance with the ethical standards of the research committee of SYSUCC. Formal written informed consent was obtained from all individual participants included in the study.

### Data collection for the TCGA cohort

Data on race, age at diagnosis, anatomic site of the tumor, histology, histologic grade, TNM stage, mutation count, therapy, and survival data in both male (*n* = 294) and female (*n* = 349) patients with a BRCA1/2 mutation (either deleterious or undetermined significance) were extracted from the MSK-IMPACT clinical sequencing cohort [18] by accessing the National Cancer Institute (NCI) Genomic Data Commons (GDC) data portal (cBioPortal; http://cbioportal.org). These data were also gathered from male patients without BRCA1/2 mutations (*n* = 4577) in this cohort. Cases with unknown sex data were excluded. All relevant data were extracted by two independent reviewers (PS and YL), and any disagreements were reviewed by a third reviewer (JHH).

### Statistical analysis

The clinicopathologic features were analyzed using SPSS software, version 26.0. Eligible patients were considered as the unmatched cohort. To reduce bias, we also developed 1:1 (168 male BRCAm carriers: 168 female BRCAm carriers; 93 male deleterious BRCAm carriers: 108 male undetermined significance BRCAm carriers) matched cohorts using propensity score matching (PSM) for age and TNM stage with a caliper of 0.01 for further survival analyses. Similarly, 1:3 (294 male BRCAm carriers: 883 non-BRCAm carriers; 111 male deleterious BRCAm carriers: 333 non-BRCAm carriers) matched cohorts were also developed. The variables were compared between the groups using the chi-square test. Overall survival (OS) and progression-free survival (PFS) curves were drawn using the Kaplan–Meier method and were compared using the log-rank test. The mutations on linear BRCA1 or BRCA2 protein and its domains were mapped using lollipop plots [19, 20]. All tests were two-sided, and a *p*-value < 0.05 was considered statistically significant.

## Results

### Demographic features

A total of 52 male Asian patients harboring BRCA mutations were identified in the SYSUCC cohort. The median age at the time of diagnosis was 63.5 years (range, 4–86 years). Most patients (36/52, 69.2%) were aged 50–69 years. Fifteen patients (28.8%) had a family history of tumor, and only 3.8% (2/52) demonstrated multiple primary tumors. The prostate was the most common site of tumor disease in this cohort (19/52, 36.5%), followed by the colorectum (13/52, 25.0%), stomach (7/52, 13.5%), and lung (4/52, 7.7%). Only one male patient with breast cancer harboring a germline BRCA mutation was observed. The majority had stage III (15/52, 28.8%) or stage IV (28/52, 53.8%) disease. Sixteen patients (30.8%) tested positive for only BRCA1 mutations, of which 3 were germline and 13 were somatic. Thirty-three (63.5%) patients harbored only BRCA2 mutations, of which 4 were germline, 26 were somatic, and 3 displayed both germline and somatic BRCA2 mutations. We also found that three patients harbored both BRCA1 and BRCA2 somatic mutations. In addition, 13.5% (7/52) of the patients showed a microsatellite instability-high (MSI-H) phenotype, while 30.7% showed moderate to high TMB (≥10 mutations/Mb). The clinical characteristics of the SYSUCC cohort are summarized in Table 1.

**Table 1:** Characteristics of male BRCAm carriers with a diagnosis of tumor in SYSUCC cohort

Variables	Male BRCAm carriers (***n*** = 52)
***Age at diagnosis (y)***	
< 40	4(7.7)
40–49	3(5.8)
50–59	15(28.8)
60–69	21(40.4)
70–79	8(15.4)
≥ 80	1(1.9)
***Family history of tumor***	
no	35(67.3)
yes	15(28.8)
unknown	2(3.8)
***Diagnose of multiple tumors***	
no	48(92.3)
yes	2(3.8)
unknown	2(3.8)
***Anatomic site of tumor***	
prostate	19(36.5)
colon/rectum	13(25.0)
stomach	7(13.5)
lung	4(7.7)
kidney	2(3.8)
head and neck	2(3.8)
breast	1(1.9)
bladder	1(1.9)
other	3(5.8)
***Histology***	
adenocarcinoma	38(73.1)
mucinous adenocarcinoma	3(5.8)
urothelial carcinoma	3(5.8)
squamous cell carcinoma	2(3.8)
undifferentiated carcinoma	2(3.8)
neuroendocrine carcinoma	2(3.8)
other	3(5.8)
***TNM stage***	
I	1(1.9)
II	6(11.5)
III	15(28.8)
IV	28(53.8)
unknown	2(3.8)
***BRCA1/2 mutation***	
BRCA1 only	16(30.8)
germline	3(5.8)
somatic	13(25.0)
both germline and somatic	0(0.0)
BRCA2 only	33(63.5)
germline	4(7.8)
somatic	26 (50.0)
both germline and somatic	3(5.7)
both BRCA1 and BRCA2	3(5.8)^a^
***MSI***	
MSS	26 (50.0)
MSI-H	7(13.5)
unknown	19(36.5)
***TMB***	
low (< 10 muts/Mb)	17 (32.7)
moderate (10–20 muts/Mb)	2(3.8)
high (> 20 muts/Mb)	14 (26.9)
unknown	19(36.5)
***Surgery***	
no	17 (32.7)
yes	34 (65.4)
unknown	1(1.9)
***Endocrinotherapy***	
no	40 (76.9)
yes	11 (21.2)
unknown	1(1.9)
***Chemotherapy***	
no	16(30.8)
yes	35(67.3)
unknown	1(1.9)
***Radiotherapy***	
no	36 (69.2)
yes	15(28.8)
unknown	1(1.9)
***Immunotherapy***	
no	44 (84.6)
yes	7(13.5)
unknown	1(1.9)
***Targeted therapy***	
no	47 (90.4)
yes	4(7.7)
unknown	1(1.9)
***Tumor progression***	
no	29 (55.8)
yes	22 (42.3)
unknown	1(1.9)
***Survival***	
alive	41 (78.9)
deceased	10 (19.2)
unknown	1(1.9)

^a^Somatic mutation in both BRCA1 and BRCA2 is found in three cases

*BRCA* Breast cancer susceptibility gene, *MSI* Microsatellite instability, *MSS* Microsatellite stable, *MSI-H* Microsatellite instability-high, *TMB* Tumor mutational burden

The incidence of BRCA mutations in male patients was 6.0% (294/4871) in the TCGA cohort, and the majority of these patients were white. The median age at the time of diagnosis was 65 years (range, 34–88 years), and the patients were predominantly 50–79 years old. Carcinomas of the lung (57/294, 19.4%), bladder (46/294, 15.6%), stomach (35/294, 11.9%), head and neck (25/294, 8.5%), and colorectum (24/294, 8.2%), as well as cutaneous melanoma (48/294, 16.3%) were frequent tumors demonstrating BRCA mutations. However, prostatic cancer was only observed in 11 cases, and three patients had breast cancer. The majority of the patients had stage II (86/294, 29.3%) or stage III (28/52, 53.8%) disease. Similar to the findings in the SYSUCC cohort, 81 patients (27.6%) had a BRCA1 mutation, while 193 patients (65.6%) had a BRCA2 mutation. Patients with both BRCA1 and BRCA2 mutations (20/294, 6.8%) showed a median total mutation count of 2390 mutations (range, 264–15,832 mutations), which was significantly higher than that in patients with either BRCA1 or BRCA2 mutations. The clinical characteristics of the TCGA cohort are summarized in Table 2.

**Table 2:** Characteristics of BRCAm carriers with a diagnosis of tumor in TCGA cohort

Variables	Male BRCAm carriers (***n*** = 294)	***Female BRCAm*** ***carriers (n = 349)***	***Male non-BRCAm*** ***carriers (n = 4577)***	P*	***P***^***#***^
***Incidence of BRCA1/2 mutation***					
carrier	294 (6.0)	349 (6.6)	–	0.275	–
non-carrier	4577 (94.0)	4969 (93.4)			
***Race***					
white	225 (76.5)	246 (70.5)	3238 (70.7)	0.001	0.316
Asian	19 (6.5)	21 (6.0)	337 (7.4)		
Black/African American	12 (4.1)	46 (13.2)	274 (6.0)		
other	0(0.0)	3 (0.9)	9 (0.2)		
unknown	38 (12.9)	33 (9.5)	719 (15.7)		
***Age at diagnosis***					
< 40	14 (4.8)	27 (7.7)	454 (9.9)	0.15	< 0.001
40–49	28 (9.5)	42 (12.0)	519 (11.3)		
50–59	64 (21.8)	93 (26.6)	1095 (23.9)		
60–69	81 (27.6)	91 (26.1)	1388 (30.3)		
70–79	81 (27.6)	68 (19.5)	858 (18.7)		
≥ 80	24 (8.2)	26 (7.4)	239 (5.2)		
unknown	2 (0.7)	2 (0.6)	24 (0.5)		
***Anatomic site of tumor***					
lung	57 (19.4)	36 (10.3)	541 (11.8)	< 0.001	< 0.001
skin	48 (16.3)	24 (6.9)	228 (5.0)		
bladder	46 (15.6)	11 (3.2)	258 (5.6)		
stomach	35 (11.9)	14 (4.0)	250 (5.5)		
head and neck	25 (8.5)	6 (1.7)	360 (7.9)		
colorectum	24 (8.2)	23 (6.6)	288 (6.3)		
kidney	14 (4.8)	8 (2.3)	565 (12.3)		
prostate	11 (3.7)	–	483 (10.6)		
CNS	8 (2.7)	9 (2.6)	452 (9.9)		
esophagus	8 (2.7)	1 (0.3)	148 (3.2)		
liver	8 (2.7)	4 (1.1)	243 (5.3)		
breast	3 (1.0)	54 (15.5)	9 (0.2)		
uterus	–	93 (26.6)	–		
ovary	–	30 (8.6)	–		
cervical	–	27 (7.7)	–		
other	7 (2.4)	9 (2.6)	752 (16.4)		
***Histology***					
adenocarcinoma	86 (29.3)	144 (41.3)	1355 (29.6)	< 0.001	< 0.001
melanoma	48 (16.3)	24 (6.9)	229 (5.0)		
urothelial carcinoma	46 (15.6)	11 (3.1)	257 (5.6)		
squamous cell carcinoma	66 (22.4)	40 (11.5)	755 (16.5)		
IBC, NST	3 (1.0)	47 (13.5)	8 (0.2)		
serous carcinoma	–	36 (10.3)	–		
other	45 (15.3)	47 (13.5)	1973 (43.1)		
***TNM stage***					
0	1 (0.3)	1 (0.3)	4 (0.1)	0.001	0.007
I	55 (18.7)	50 (14.3)	1025 (22.4)		
II	86 (29.3)	82 (23.5)	864 (18.9)		
III	72 (24.5)	40 (11.5)	823 (18.0)		
IV	39 (13.3)	8 (2.3)	507 (11.1)		
unknown	41 (13.9)	168 (48.1)	1354 (29.6)		
***BRCA1/2 mutation***					
BRCA1 only	81 (27.6)	117 (33.5)	–	0.001	–
BRCA2 only	193 (65.6)	183 (52.4)			
both BRCA1 and BRCA2	20 (6.8)	49 (14.1)			
***Histologic grade***					
low	8 (2.7)	18 (5.2)	132 (2.9)	0.309	0.875
moderate to high	122 (41.5)	170 (48.9)	1774 (38.8)		
unknown/unclassified	164 (55.8)	160 (46.0)	2671 (58.4)		
***Mutation count (median)***					
all	322 (range, 11–15,832)	379 (range, 7–25,730)	63(range, 1–5865)	–	–
with BRCA1 mutation only	309 (range, 24–3679)	161 (range, 8–5118)	–		
with BRCA2 mutation only	266 (range, 11–6369)	340 (range, 7–12,071)	–		
with BRCA1 & BRCA2 mutation	2390 (range, 264–15,832)	8234 (range, 153–25,730)	–		
***Radiotherapy***					
no	215 (73.1)	218 (62.4)	2920 (63.8)	< 0.001	0.009
yes	48 (16.3)	98 (28.1)	1010 (22.1)		
unknown	31 (10.6)	33 (9.5)	647 (14.1)		
***Neoadjuvant therapy***					
no	290 (98.6)	348 (99.7)	4523 (98.8)	0.274	0.782
yes	4 (1.4)	1 (0.3)	54 (1.2)		
***Tumor progression***					
no	168 (57.1)	258 (73.9)	2865 (62.6)	< 0.001	0.078
yes	125 (42.5)	90 (25.8)	1707 (37.3)		
unknown	1 (0.3)	1 (0.3)	5 (0.1)		
***Overall survival***					
alive	172 (58.5)	282 (80.8)	3149 (68.8)	< 0.001	< 0.001
deceased	121 (41.2)	66 (18.9)	1423 (31.1)		
unknown	1 (0.3)	1 (0.3)	5 (0.1)		

* χ^2^ test comparing proportions between male and female BRCA1/2 mutation carriers, unknown or missing data is excluded from χ^2^ test

^**#**^ χ^2^ test comparing proportions between male patients with and without BRCA1/2 mutation, unknown or missing data is excluded from χ^2^ test

*BRCA* Breast cancer susceptibility gene, *IBC* Invasive breast carcinoma, *NST* Non-special type, *CNS* Central nervous system

### Clinical characteristics of males and females with BRCAm

**Fig. 1:**
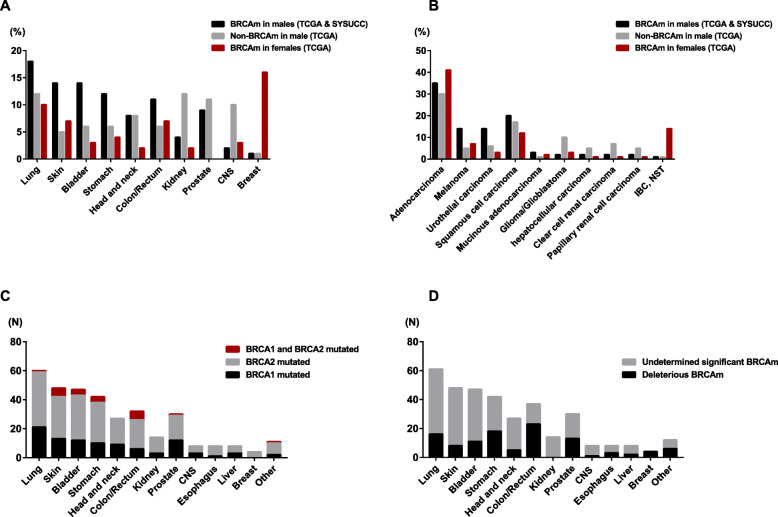
The characteristics of BRCA-associated tumors in males. The anatomic sites (**a**) and histological types (**b**) of tumors occurring in male and female patients with BRCAm as well as non-BRCAm male carriers. **c**, **d** Mutation variants of BRCA1/2 in BRCA-associated tumors in males

The clinical characteristics were compared between male and female patients with BRCAm, as shown in Table 2. The incidence of BRCAm (6.0 vs. 6.6%) as well as the age at diagnosis of tumor were similar between male and female patients, and no significant difference was observed (*p* = 0.275 and *p* = 0.15, respectively). However, the spectrum of tumors that occurred in males was obviously different from that in females. Carcinomas of the breast, ovary, uterus, and cervix frequently occurred in females with BRCAm, while a higher incidence of carcinomas in the lung, bladder, stomach, and head and neck, as well as cutaneous melanoma, was found in male BRCAm carriers (Fig. 1a). Adenocarcinoma was the most common histological type in both groups, while there were more cases with melanoma, urothelial carcinoma, and squamous cell carcinoma in males (Fig. 1b). In addition, stage III (24.5 vs. 11.5%) and stage IV (13.3% vs. 2.3%) disease were more common in males than in females. Interestingly, individuals with both BRCA1 and BRCA2 mutations were more frequently observed in females (14.1% vs. 6.8%).

### Clinical characteristics of males with and without BRCAm

The mean age of male patients without BRCAm at the time of diagnosis was 61 years (range, 14–86 years), which was younger than that of male patients with BRCAm (*p* < 0.001). No difference was observed in the ethnic distribution between groups. Notably, compared to non-BRCAm carriers, cutaneous melanoma (16.3 vs. 5.0%), lung cancer (19.4 vs. 11.8%), bladder cancer (15.6 vs. 5.6%), and stomach cancer (11.9 vs. 5.5%) accounted for a significantly higher proportion of the tumors diagnosed in the BRCAm group. There were fewer patients that had a diagnosis of renal cell carcinoma (4.8 vs. 12.3%) or prostatic carcinoma (3.7 vs. 10.6%) in the BRCAm group than in the non-BRCAm group (Table 2, Fig. 1a). Histologically, adenocarcinoma was the most common type in both groups, but melanomas and urothelial carcinomas were more commonly found in the BRCAm group (Fig. 1b). Patients with BRCAm were also likely to present a more advanced stage of disease (*p* = 0.007) and higher total mutation counts than those without BRCAm (median, 322 vs. 63 mutations).

### The mutational profiles of BRCA1/2 in males

The characteristics of BRCA1/2 mutations in male and female patients with a diagnosis of cancer are detailed in Table 3. A total of 438 BRCA1/2 variations were identified in the males in the TCGA cohort (details provided in Supplementary table 1) and SYSUCC cohort (details provided in Supplementary table 2). Mutations affecting BRCA1 and BRCA2 accounted for 29.0% (127/438) and 71.0% (311/438), respectively. For BRCA1, 21.8% (29/127) of the mutations were deleterious or likely deleterious. Similarly, 28.6% (89/311) of BRCA2 variations were identified as deleterious or likely deleterious. More than one BRCA1/2 mutation was observed in 17.3% (60/346) of the male patients. Missense mutations were the predominant mutational types of both BRCA1 and BRCA2, accounting for 70.1% (89/127) and 69.5% (216/311), respectively, followed by nonsense and frameshift mutations.

**Table 3:** Mutational profiles of BRCA1/2 in male and female patients with a diagnosis of tumor

Variables	BRCAm identified in males (***n*** = 438, TCGA & SYSUCC)	BRCAm identified in ***females (n = 592, TCGA)***	P*
***Oncogenicity of BRCA1/2 mutation***			
BRCA1	***127 (29.0)***	***193 (32.6)***	***0.243***
deleterious (or likely)	29 (21.8)	67 (34.7)	0.032
undetermined significant	98 (77.2)	126 (65.3)	
BRCA2	***311 (32.6)***	***399 (67.4)***	
deleterious (or likely)	89 (28.6)	103 (25.8)	0.454
undetermined significant	222 (71.4)	296 (74.2)	
***Individual BRCA1/2 mutation count*** ***(range, 1–12)***			
1	286 (82.7)	278 (79.7)	0.208
2	36 (10.4)	32 (9.2)	
≥ 3	24 (6.9)	39 (11.2)	
***BRCA1/2 mutational types***			
BRCA1			
frameshift	7 (5.5)	20 (10.4)	0.013
missense	89 (70.1)	144 (74.6)	
nonsense	15 (11.8)	23 (11.9)	
in-frame InDel	2 (1.6)	0(0.0)	
fusion/amplification	5 (3.9)	3 (1.6)	
others^a^	9 (7.1)	3 (1.6)	
BRCA2			
frameshift	47 (15.1)	38 (9.5)	0.110
missense	216 (69.5)	304 (76.2)	
nonsense	33 (10.6)	44 (11.0)	
in-frame InDel	2 (0.6)	3 (0.8)	
fusion/amplification	2 (0.6)	4 (1.0)	
others	11 (3.5)	6 (1.5)	

*χ^2^ test comparing the characteristics of BRCA1/2 mutations between groups

^a^Including BRCA1/2 intronic mutations in splice site (or region) and translation start site

As shown in Fig. 1c, BRCA2 mutation was more frequently detected than BRCA1 mutation regardless of the location of the tumor in males and accounted for 56.7–100.0% of the cases. Mutations simultaneously affecting both BRCA1 and BRCA2 mainly occurred in head/neck squamous cell carcinoma (18.8%) and cutaneous melanoma (12.5%). However, in terms of pathogenicity (Fig. 1b), only a few BRCA1/2 mutations in cutaneous melanoma (8/48, 16.7%) and tumors located in the head and neck (5/27, 18.5%) and CNS (1/7, 12.5%) were deleterious. None of the BRCA1/2 mutations in the renal cell carcinoma cases included in this study were deleterious (0/14). More frequent deleterious mutations were identified in carcinomas of the colorectum (23/37, 62.2%), prostate (13/30, 43.3%), and stomach (18/42, 42.9%). Notably, all BRCA mutations in male breast cancer were deleterious (4/4). Compared to BRCA1/2 mutations in female patients, fewer deleterious (or likely deleterious) mutations affecting BRCA1 were detected in males (21.8 vs. 34.7%, *p* = 0.032), while those affecting BRCA2 showed no difference between groups (28.6 vs. 25.8%, *p* = 0.454). In addition, male patients displayed fewer frameshift mutations in BRCA1 than female patients (5.5 vs. 10.4%, *p* = 0.013). In contrast, frameshift mutations in BRCA2 were likely more frequently found in males (15.1% vs. 9.5%), while no significant difference was observed.

**Fig. 2:**
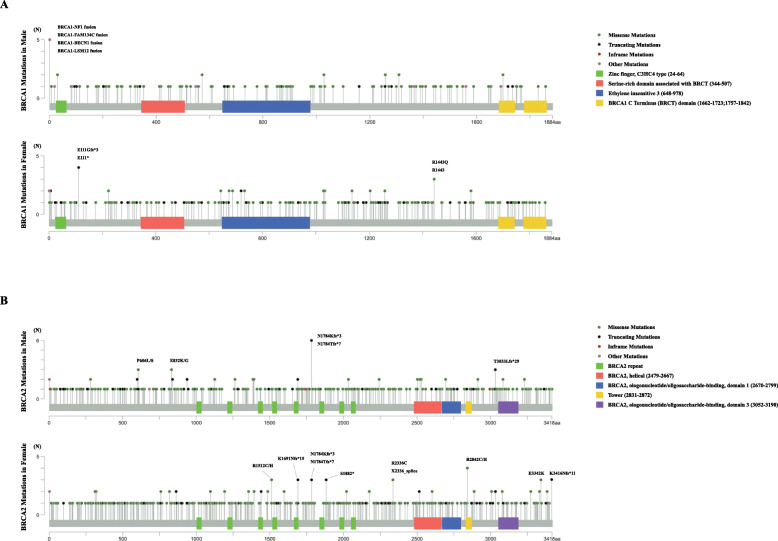
Locations of mutations in the linear BRCA1 and BRCA2 proteins. The X-axis represents the amino acid (aa) residues of the BRCA1 or BRCA2 protein, and the Y-axis represents the mutation frequencies. **a** BRCA1 mutations identified in male and female patients. **b** BRCA2 mutations detected in male and female patients. Mutations repeated three times or more are highlighted

Mutations on the linear BRCA1 or BRCA2 protein and its domains were mapped as shown in Fig. 2. In accordance with the findings of previous studies, there was no obvious hotspot mutational region in either BRCA1 or BRCA2. In male BRCA1 mutation carriers, only 2 of 127 mutations (1.6%) were located in the RING domain that associates with BRCA1-associated RING domain protein 1 (BARD1) and catalyzes protein ubiquitylation. Eight mutations (6.3%) were found either at the BRCA1 C-terminus (BRCT) domain or at the serine-rich domain associated with BRCT, which facilitates phospho-protein binding, checkpoint activation and DNA repair. Similarly, in female BRCA1 mutation carriers, 3.1% (6/193) of mutations were located in the RING domain. In addition, 6.2% (12/193) and 4.7% (9/193) were mutated at the BRCT domain and serine-rich domain associated with BRCT, respectively. BRCA1 fusion mutations containing preferred partners, including NF1, FAM134C, BECN1, and LSM12, were more frequently detected in male carriers, while mutations affecting the E111 (E111*, E111Gfs*3) or R1443 (R1443*, R1443Q) sites were more commonly found in BRCA1 in female patients. In male BRCA2 mutation carriers, a total of 19 mutations (6.1%) were detected in the eight centrally located BRC repeats in BRCA2, which have been suggested to mediate the binding and regulation of RAD51 on resected DNA substrates. We also found 20.3% (63/311) of the mutations located in the BRCA2 DNA-binding domain, of which 5.1% (16/311) were in the helical domain, 14.2% (44/311) were in oligonucleotide binding folds, and 1.0% (3/311) were in the tower domain, which may affect BRCA2 binding to both single-stranded DNA and double-stranded DNA. In female BRCA2 mutation carriers, 6.3% (25/399) of the mutations affected BRC repeats, and 18.3% were located in the BRCA2 DNA-binding domain, of which 5.0% (20/399) were located in the helical domain, 11.3% (45/399) were located in oligonucleotide binding folds, and 2.6% (8/311) were located in the tower domain. In addition, frameshift mutations in BRCA2 at N1784 (N1784Kfs*3, N1784Tfs*3) were frequently observed in both male and female patients. Recurrent BRCA2 mutations at P606L/S, E832K/G, and T3033Lfs*29 were shown in males, while those at R1512C/H, K1691Nfs*15, S1882*, R2842C/H, E3342K, and K3416Nfs*11 appeared repeatedly in female BRCA2 mutation carriers.

### Prognostic implications of BRCAm in males

**Fig. 3:**
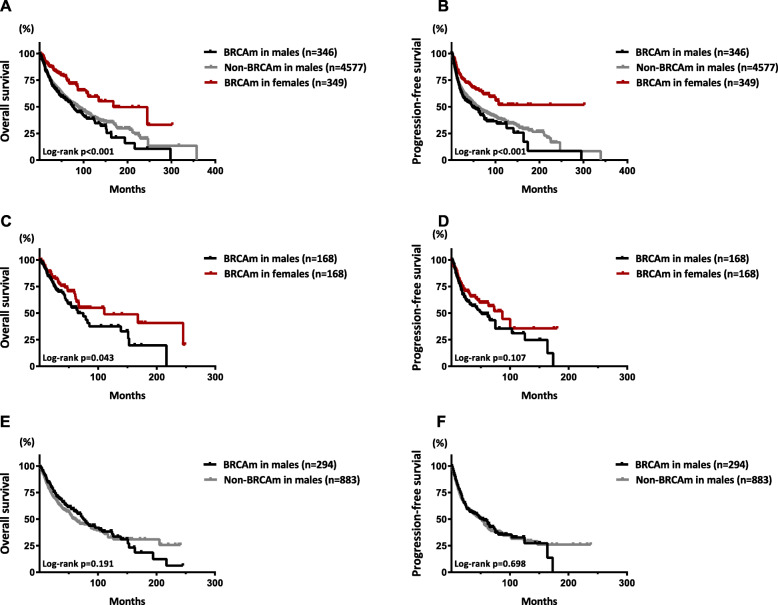
Survival curves of overall survival and progression-free survival in male patients with BRCA-associated tumors. Kaplan-Meier curves of OS (**a**) and PFS (**b**) among male and female patients with BRCAm as well as non-BRCAm male carriers in the unmatched cohort. Kaplan-Meier curves of OS (**c**) and PFS (**d**) between male and female patients with BRCAm in a 1:1 matched cohort. Kaplan-Meier curves of OS (**c**) and PFS (**d**) between male patients with and without BRCAm in a 1:3 matched cohort. Propensity score matching for age and TNM stage with a caliper of 0.01 was performed to establish the matched cohorts

**Fig. 4:**
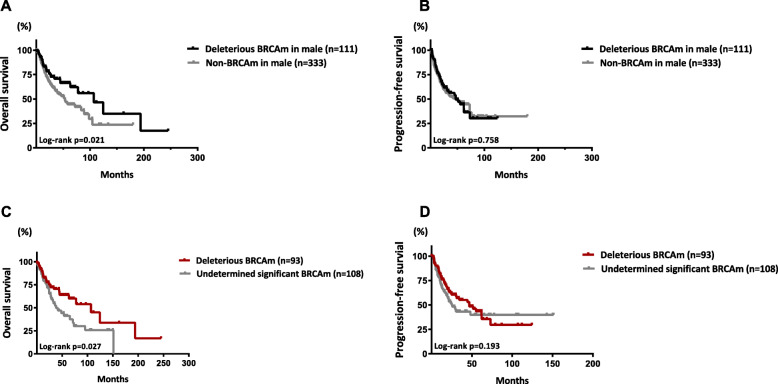
Survival curves of overall survival and progression-free survival among male patients with deleterious BRCA mutations. Kaplan-Meier curves of OS (**a**) and PFS (**b**) between male patients with deleterious BRCAm and those without BRCAm in a 1:3 matched cohort. Kaplan-Meier curves of OS (**c**) and PFS (**d**) between male patients with deleterious BRCAm and those with BRCAm of undetermined significance in a 1:1 matched cohort. Propensity score matching for age and TNM stage with a caliper of 0.01 was performed to establish the matched cohorts

Male BRCAm carriers with a diagnosis of malignancy were followed-up for 0.3–151.3 months, with a median of 25.9 months. In comparison, male patients without BRCAm were followed-up for 0.1–357.4 months, with a median of 22.9 months. Moreover, female patients with BRCAm were followed-up for 0.1–302.1 months, with a median of 26.2 months. Survival analyses were conducted in both the unmatched and matched cohorts. The survival curves of the three groups are shown in Fig. 3. BRCAm in males was associated with a decrease in overall survival (OS) and progression-free survival (PFS) when compared to female patients with BRCAm in both the unmatched and matched cohorts (Fig. 3a-d; *p* < 0.001). No significant difference in OS was observed between male patients with and without BRCAm in either the unmatched (*p* = 0.698; Fig. 3a) or matched cohort (*p* = 0.191; Fig. 3e), but those with deleterious BRCAm displayed a significantly increased OS compared with non-BRCAm carriers in both the 1:3 (*p* = 0.021; Fig. 4a) and 1:6 matched models (*p* = 0.042; Supplementary figure 1). Furthermore, among male patients with BRCAm, after matching for age and TNM stage, those with deleterious BRCAm also displayed an increased OS compared with patients with BRCAm of undetermined significance (*p* = 0.027; Fig. 4c). However, no difference in PFS was found in any comparisons between groups in males (Fig. 3b, f and Fig. 4b, and d). Subgroup survival analysis was also conducted among male patients with either BRCA1, BRCA2 or both two mutations, but no difference in survival was found (Supplementary figure 2).

**Fig. 5:**
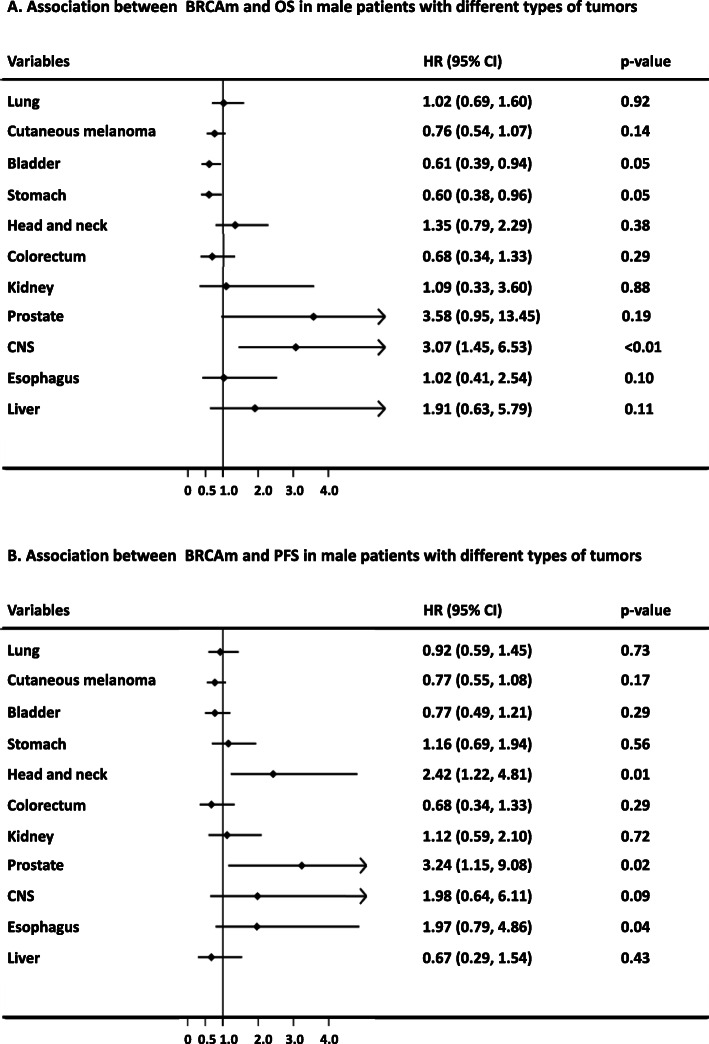
The prognostic implications of BRCAm in male patients with different types of tumors. Association between BRCAm and OS (**a**) or PFS (**b**) in male patients with different types of tumors

**Fig. 6:**
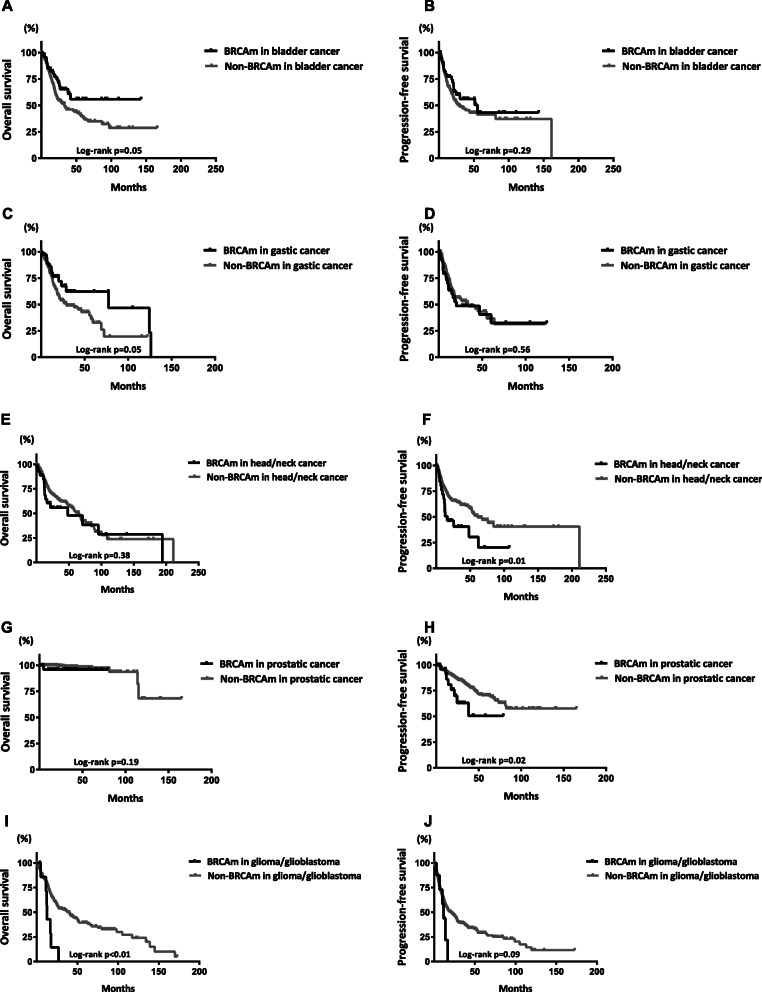
Survival curves between male patients with and without BRCAm in the subgroups of different tumors. Survival curves of OS and PFS in the subgroups of bladder cancer (**a**, **b**), gastric cancer (**c**, **d**), head and neck cancer (**e**, **f**), prostatic cancer (**g**, **h**), and glioma or glioblastoma (**i**, **j**)

The associations between BRCAm and the survival outcomes of male patients in the subgroups of different tumor types are shown in Fig. 5. BRCAm was associated with hazard ratios for OS of 0.61 (95% CI 0.39–0.94; *p* = 0.05) and 0.60 (95% CI 0.39–0.94; *p* = 0.05) in the subgroups of patients with bladder cancer and stomach cancer, respectively. In contrast, the hazard ratio for OS was 3.07 (95% CI 1.45–6.53; *p* < 0.01) in glioma/glioblastoma patients with BRCAm. In addition, BRCAm was associated with hazard ratios for PFS of 2.42 (95% CI 1.22–4.81; *p* = 0.01) and 3.24 (95% CI 1.15–9.08; *p* = 0.02) in patients with prostatic cancer and in those with head and neck squamous cell carcinoma, respectively. The survival curves of OS and PFS between the groups of these tumors are shown in Fig. 6.

## Discussion

Previous epidemiologic studies have demonstrated that BRCA mutation carriers have a high risk of developing various tumors later in life [4–10]. The reported risks for breast, prostate, pancreatic, and colorectal cancers are higher in male BRCA mutation carriers than in noncarriers [7–10]. However, little is known about the tumor spectrum, clinicopathologic features, prognosis, and BRCA mutational profiles of male patients with BRCA-associated tumors. To the best of our knowledge, the present study is the first retrospective analysis of a large cohort of BRCA-associated pan-tumors in males. We also provide an overview of the differences in tumor characteristics and prognoses between BRCAm and non-BRCAm carriers, aiming to raise more concern about genetic BRCA testing for males.

Our findings revealed that the incidence of BRCAm in patients with pan-tumors was actually similar (~ 6.0–6.6%) between males and females. BRCA2 mutations were more commonly detected than BRCA1 mutations in both male and female patients, which is consistent with previously reported data [4, 21–23]. Although distinct differences may exist depending on populations, races, and tumor types [24], Marabelli et al. [25] reported that far fewer men than women (less than 1:10) were tested for BRCA1/2 mutations in European countries and that broader genetic data on males may contribute to the diagnosis and management of patients and increase treatment and clinical trial options. The frequency of BRCA mutations varied considerably in males. Previous studies reported that 4–40% and 0–11% of patients with male breast cancer harbored BRCA2 and BRCA1 mutations, respectively [26–28]. Several other groups have examined the incidence of deleterious BRCA mutations in unselected series of patients with prostate cancer, which was approximately 1.4–5.7% [29, 30]. Murphy et al. [31] and Goggins et al. [7, 32] reported a relatively high incidence of deleterious BRCA2 mutations in pancreatic cancer. Similar findings on the incidence of BRCA mutations were observed in breast cancer (25.0%) in our study, while the incidence was slightly higher (2.2%) in prostate cancer. Only 3 of 153 (1.9%) male patients with pancreatic cancer had BRCAm. In addition, neither prostate cancer nor pancreatic cancer was among the common tumor types of patients with BRCAm, while more attention to BRCA mutations should also be raised in individuals with cutaneous melanoma (17.4%), bladder cancer (15.1%), gastric cancer (12.3%), and lung cancer (9.5%). On the other hand, we also found that more frequent deleterious variants were identified in carcinomas of the breast, colorectum, prostate, and stomach, which indicated that BRCAm may act as a potential driver mutation in these tumors in males.

The most recent NCCN guidelines recommend BRCA testing for male breast cancer patients, metastatic or advanced prostate cancer patients and those with a family history of the disease. Younger carriers and those with a family history or multiple tumors were reported to be more likely to carry BRCA1/2 mutations [7, 33–36]. However, the definition of younger age may be different in various tumors or between sexes. We observed that the median age of female patients with BRCAm was younger than that of male carriers in our cohort (60 vs. 65 years), which may be because the common tumors that occurred in female BRCAm carriers were predominantly estrogen-related, including breast, ovarian, and uterine cancer. Some studies revealed that male carriers younger than 65 years were at higher risk for prostate, pancreatic, and colon cancer [37], while another report showed a cut-off value of 73 years [38]. Ibrahim et al. [36] conducted a pan-tumor study in males and demonstrated that over one-third of BRCA2 mutation carriers had multiple tumors. They suggested that physicians should be vigilant to the synchronous or metachronous development of a secondary cancer in BRCAm carriers. However, only 1 of 52 patients in the SYSUCC cohort displayed multiple tumors (synchronous colonic and lung cancers). Over 28.8% of patients with BRCAm in the SYSUCC cohort had a family history of cancer, but only two of these patients were identified as having germline mutations. Seven patients had the same cancer as the family history, including cancer of the colon (three patients), nasopharynx (two patients), stomach (one patient), and prostate (one patient). BRCAm was incidentally detected by multigene panel testing in up to 65.4% of the cases. We suggest that male patients with a family history of cancer or multiple tumors should be considered for BRCA testing. Those with older age or metastatic/advanced cancer of the bladder, colon, stomach, lung, or pancreas, as well as cutaneous melanoma might also be included, though more relevant clinical evidence and criteria are needed.

The prognostic implication of BRCA-associated tumors remains a controversial issue [7–9, 39–42]. The pan-tumor analysis in this study showed decreased OS and PFS in male patients compared with female patients with BRCA-associated tumors, which may principally be due to the differences in tumor types and the corresponding available treatment options. Histopathological studies revealed that BRCA-associated tumors were usually high grade, poorly differentiated, and accompanied by a higher frequency of somatic abnormalities [9, 43, 44], which was also observed in our study. Along with the general nature of BRCA functions, these findings might logically support that BRCAm carriers tend to have a worse prognosis than noncarriers. However, we failed to demonstrate distinct PFS or OS between BRCA-associated and non-BRCA-associated tumors in males. Increased OS was found in the deleterious BRCAm group compared to the non-BRCA-associated group or the BRCAm of undetermined significance group. Existing clinical studies have also suggested that some BRCA-associated tumors are more sensitive to agents targeting DNA repair pathways, such as systemic chemotherapy (platinum-based) or PARPi [11–15]. However, the better response to these systemic treatments might not necessarily translate into better clinical outcomes in BRCAm carriers [11–15]. The subgroup survival analysis indicated that the prognostic value of BRCAm may vary in different tumors. We found for the first time that BRCAm was associated with increased OS in male patients with gastric or bladder cancer. There are currently limited data regarding the prognosis of BRCA mutation carriers. A study by Halpern et al. [45] reported a relatively favorable prognosis in seven BRCA-associated metastatic gastric cancer patients treated with DNA-damaging agents. Another study by Nickerson et al. [46] identified frequent BRCA1-associated protein-1 (BAP1) and BRCA pathway alterations in bladder cancer. Consistent with the reported data [9, 41, 42, 44], worse survival was displayed in BRCAm carriers with prostate cancer in this cohort. Altered BRCA pathways have also been implicated in head and neck squamous cell carcinoma, esophageal carcinoma, and glioma/glioblastoma. Recent studies suggest that the activity of the Fanconi anemia/BRCA pathway may predict the cisplatin response in head and neck squamous cells [47, 48]. Secrier et al. [49] identified an enriched BRCA mutational signature with prevalent defects in the homologous recombination pathway in some esophageal adenocarcinoma cases. Isolated cases of glioma with BRCAm were also reported [50]. In the present study, we first found that BRCAm may also be associated with decreased PFS in male patients with cancer of the esophagus or head and neck, as well as decreased OS in those with glioma/glioblastoma, suggesting that more BRCA testing and the corresponding investigations regarding the risk, prognosis and treatment options of the diseases should be further conducted in patients with these tumors.

On the other hand, we also noticed that seven patients in the SYSUCC cohort with MSI-H and high TMB were deleterious BRCA2 variant carriers with colon, prostate, nasopharyngeal, or gallbladder cancer. All of them had received immune checkpoint inhibitors (pembrolizumab, etc.). Only one patient died of the tumor. In addition, one patient with stage IV colon cancer carrying a deleterious variant of somatic BRCA2 mutation received maintenance PARPi using olaparib and had stable disease for 7.8 months. Studies by McAlpine et al. [51] and Clarke et al. [52] have found that BRCA mutations may be correlated with immunogenicity in ovarian high-grade serous carcinoma. Most importantly, the available preclinical and translational data strongly support that the DNA damage and tumor cell death caused by PARPi may have the potential to reconstitute the immune microenvironment of tumors and imply the clinical value of combining the PARP inhibition and immune checkpoint blockade strategies [53]. More solid evidence is expected from the data of ongoing [54, 55] and further clinical trials.

One limitation of the present study was the small number of cases and limited source location of samples. The inextricable selection bias in both cohorts may lead to a less generalized conclusion. For example, few patients with certain tumor types, such as liver cancer, esophageal cancer, and CNS tumors, were included in the survival analysis and had a relatively short median follow-up time. The inclusion of a larger cohort is crucial and could offer more practical insights. In addition, data on germline BRCA mutations and individual clinical treatments, such as chemotherapy, immunotherapy, and PARPi, were only identified in 52 patients in the SYSUCC cohort, while these data were not available in the remaining cases obtained from the TCGA cohort, which may lead to a less evidential conclusion.

## Conclusion

The present study is the first to provide a pan-tumor survey on BRCA-associated tumors in males. Our findings demonstrated a similar incidence of BRCA-associated tumors in male and female patients. Male BRCA-associated tumors were predominantly stage II or III disease with a higher frequency of BRCA2 mutations and were more commonly found in cutaneous melanoma and carcinoma of the lung, bladder, and stomach compared to those in non-BRCAm carriers. Frequent BRCA1 fusions and BRCA2 mutations at P606L/S, E832K/G, and T3033Lfs*29 were detected in male BRCA-associated tumors. Male patients with deleterious BRCAm generally show a better prognosis than non-BRCAm carriers. However, the prognostic implications of BRCAm varied in different tumor types. These findings have provided evidence that male BRCA-associated tumors could be clinically and genetically distinct from those in females, suggesting more BRCA testing in males and further investigations concerning the risk and prognosis associated with certain tumors, such as gastric, bladder, esophageal, and head and neck cancer, as well as glioma/glioblastoma in males. Further clinical evidence on potential treatments, including platinum-based chemotherapy, immunotherapy, PARPi, etc., is expected for these tumors.

## Supplementary information

**Additional file 1: Supplementary Figure S1**. Survival curves of overall survival (A) and progression-free survival (B) between deleterious BRCAm and non-BRCAm carriers in males. A 1:6 propensity score matching for age and TNM stage with a caliper of 0.01 was performed to establish the matched cohorts.

**Additional file 2: Supplementary Figure S2**. Survival curves of overall survival (A) and progression-free survival (B) among male patients with BRCA1, BRCA2, and both mutations.

**Additional file 3: Supplementary Table S1.** Characteristics of BRCA1/2 mutations detected in TCGA cohort.

**Additional file 4: Supplementary Table S2.** Characteristics of BRCA1/2 mutations detected in SYSUCC cohort.

## Data Availability

The datasets generated and/or analysed during the current study are also available from the corresponding author on reasonable request.

## Abbreviations

BRCA: Breast cancer susceptibility gene
BRCAm: BRCA1/2 mutation
PARPi: Poly ADP ribose polymerase inhibitor
MSI: Microsatellite instability
TMB: Tumor mutation burden
OS: Overall survival
CNS: Central nervous system
PFS: Progression-free survival
PSM: Propensity score matching
TCGA: The cancer genome atlas
