# Supplementary figures and images for: Clinical characteristics and prognostic implications of BRCA-associated tumors in males: a pan-tumor survey
Source: BMC Cancer. 2020 Oct 14;20:994. doi: 10.1186/s12885-020-07481-1 (PMC7556962; doi:10.1186/s12885-020-07481-1)

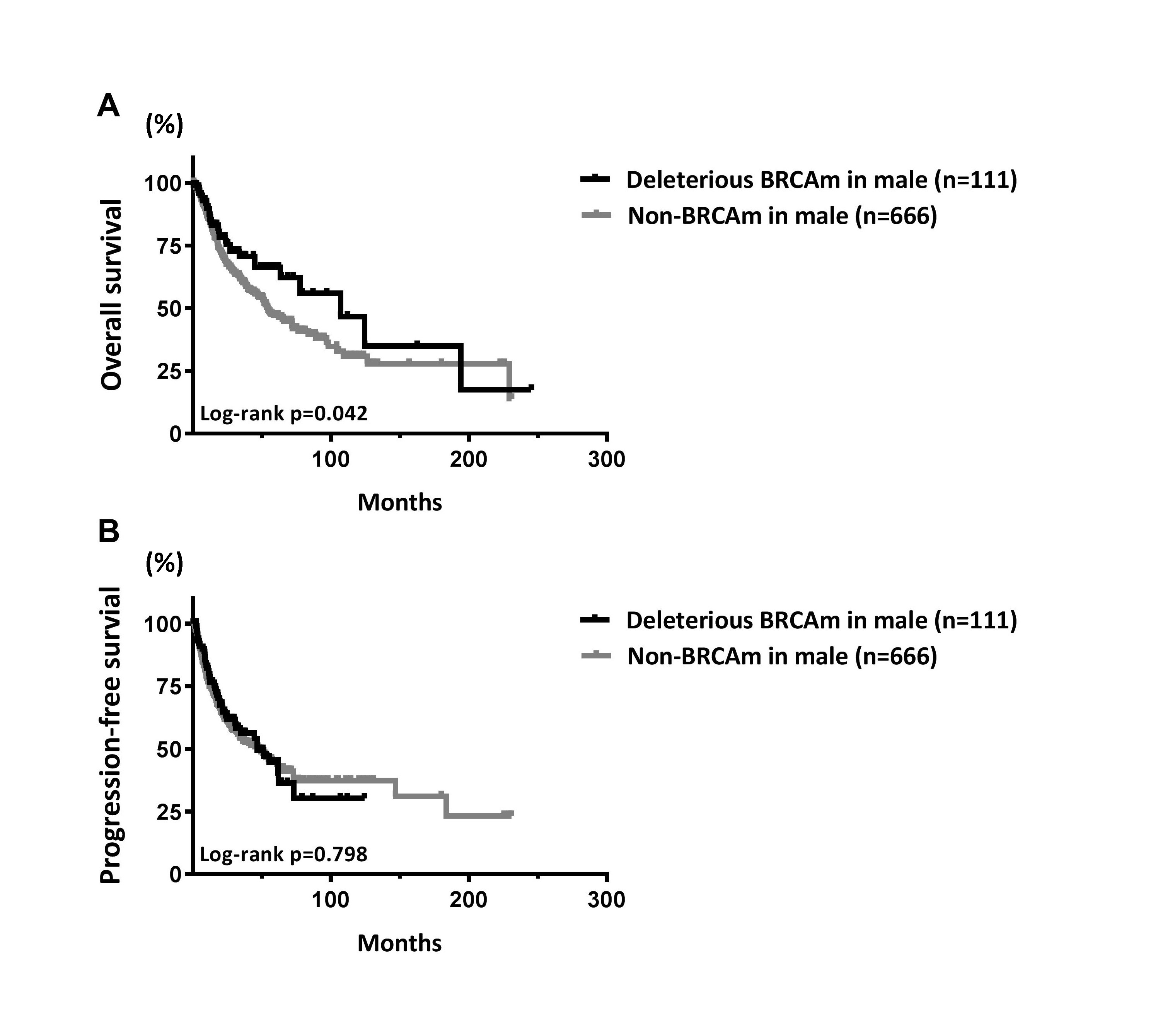

Supplement: Supplementary file 1 — Additional file 1: Supplementary Figure S1. Survival curves of overall survival (A) and progression-free survival (B) between deleterious BRCAm and non-BRCAm carriers in males. A 1:6 propensity score matching for age and TNM stage with a caliper of 0.01 was performed to establish the matched cohorts. [file 12885_2020_7481_MOESM1_ESM.jpg]

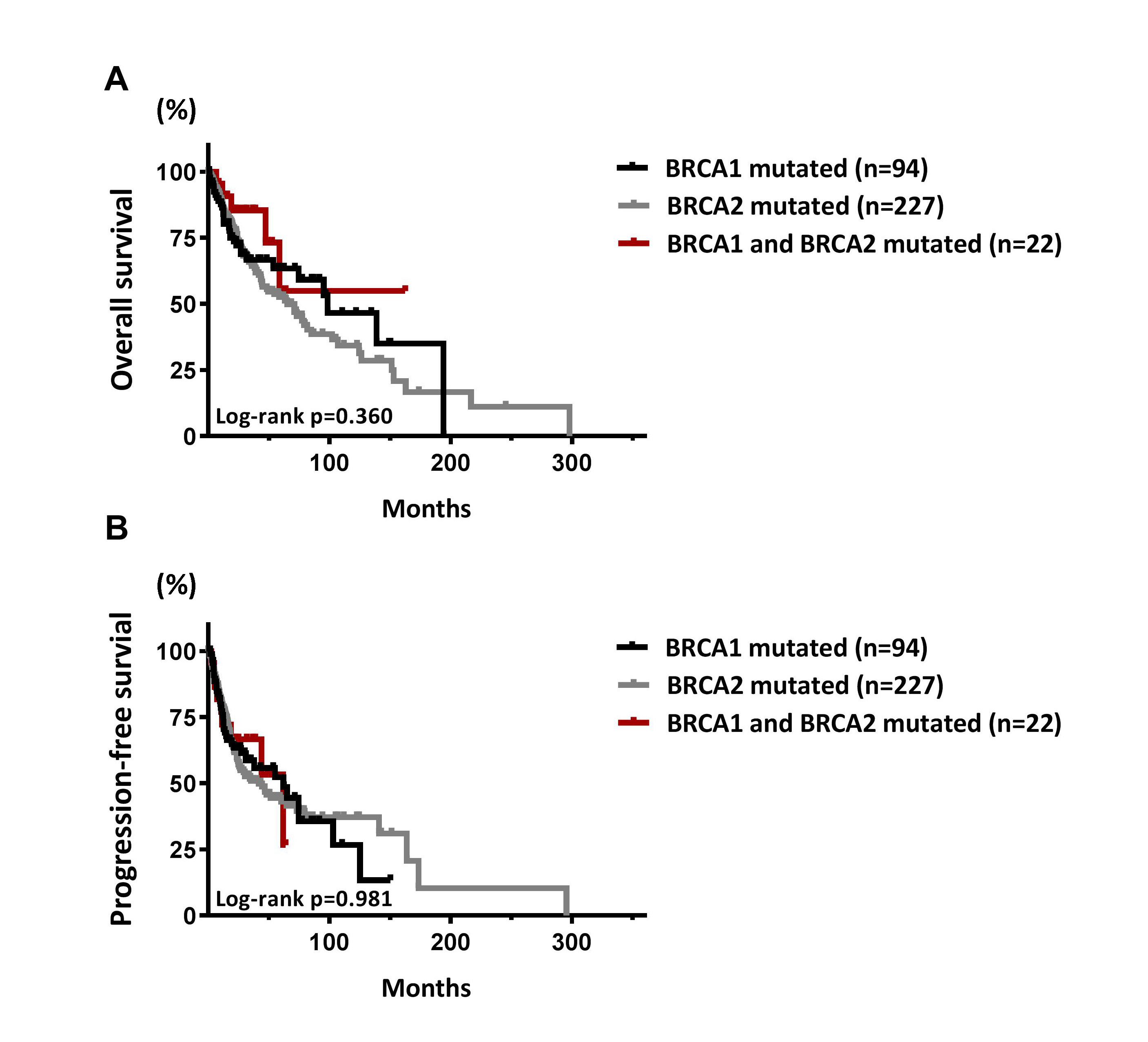

Supplement: Supplementary file 2 — Additional file 2: Supplementary Figure S2. Survival curves of overall survival (A) and progression-free survival (B) among male patients with BRCA1, BRCA2, and both mutations. [file 12885_2020_7481_MOESM2_ESM.jpg]
